# The Criterion Validity of a Newly Developed Ballroom Aerobic Test (BAT) Protocol Against Objective Methods

**DOI:** 10.3390/sports13100337

**Published:** 2025-10-01

**Authors:** Tamara Despot, Davor Plavec

**Affiliations:** 1 Faculty of Kinesiology, University of Zagreb, 10 000 Zagreb, Croatia; 2 Dance Studio ‘Dancespot’, 10 000 Zagreb, Croatia; 3 Facility for Medical Care ‘Prima Nova’, 10 000 Zagreb, Croatia; davor.plavec@primanova.hr; 4 Medical Faculty Osijek, JJ Strossmayer University, 31 000 Osijek, Croatia

**Keywords:** dance, field-based test, aerobic capacity, agreement, Bruce protocol, KF1 protocol, MetaMax^®^ 3B portable gas analyzer

## Abstract

Although laboratory testing to assess aerobic capacity has been a ‘gold standard’ in sports science, its high costs and time-consuming protocols may not be feasible for monitoring and tracking progress in limited conditions. In dancesport athletes, several field-based aerobic tests have been proposed, but the majority of them have been developed for ballet or contemporary dancers at the individual level, while the data among dance couples engaging in standard dance styles is lacking. Therefore, the main purpose of this study was to validate a newly developed Ballroom Aerobic Test (BAT) protocol against objective methods. Twelve standard dancesport couples (age: 20.4 ± 3.9 years; height: 172.1 ± 8.7 cm; weight: 60.1 ± 9.4 kg) with 8.2 ± 3.4 years of training and competing experience participated in this study. Ventilatory and metabolic parameters were generated using the MetaMax^®^ 3B portable gas analyzer (the BAT), while the KF1 (an increase in speed by 0.5 km * h^−1^ by every minute) and Bruce protocols were followed in laboratory-based settings on the running ergometer. Large to very large correlations were obtained between the BAT and KF1/Bruce protocols for the absolute maximal oxygen uptake (VO_2_max; *r* = 0.88 and 0.87) and relative VO_2_max (*r* = 0.88 and 0.85), respiratory exchange ratio (RER; *r* = 0.78 and 0.76), expiratory ventilation (VE; *r* = 0.86 and 0.79), tidal volume (VT; *r* = 0.75; 95% CI = 0.57–0.87; *p* < 0.001), ventilatory equivalent for O_2_ (VE/VO_2_; *r* = 0.81 and 0.80) and CO_2_ (VE/VCO_2_; *r* = 0.78 and 0.82), and dead space (VD/VT; *r* = 0.70 and 0.74). The Bland–Altman plots indicated no systematic and proportional biases between the BAT and KF1 protocols (standard error of estimate; SEE = ± 3.36 mL * kg^−1^ * min^−1^) and the BAT and Bruce protocols (SEE = ± 3.75 mL * kg^−1^ * min^−1^). This study shows that the BAT exhibits satisfactory agreement properties against objective methods and is a valid dance protocol to accurately estimate aerobic capacity in dancesport athletes participating in standard dance styles.

## 1. Introduction

Dance has often been defined as a combination of art and sport, with some intermittent intensity elements which are performed at higher levels [1]. Regardless of the style of dance, the ability to withstand the training and competition schedules is a prerequisite, and these physiologic capabilities are essential. Evidence suggests that aerobic capacity plays a significant role for success in dance [2]. Prioritizing the efficiency of the uptake, transport, and oxygen use efficiently is crucial for dance performance [3]. However, it has been noted that both semi-professional and professional dancers tend to have lower levels of maximal oxygen uptake (VO_2_max) in comparison to other athletes [4]. The VO_2_max in dancesport athletes is approximately 30% lower, in comparison to mono-structural and team sport athletes [1]. A further explanation for the VO_2_max hypothesis in dance might relate to the limited capacity of dance to stimulate positive training adaptations. Research has shown that improvements in aerobic capacity are not linked to rehearsal activities but rather to the intensity and duration of physical performances [5]. During specific dance movements or training protocols, the level of VO_2_max should ideally be maintained at a level between 70% and 80% of an individual’s VO_2_max, and the duration should exceed 20 min in order to effectively boost the aerobic capacity [1]. From a practical view, complex dance movements do not necessarily mean intensity but rather complexity. The shortcoming of dance is that there is no methodology for developing aerobic capacity and defined dance structures through which both intensity and duration are achieved. Thus, aerobic endurance is maintained or even increased through multiple repetitions of dance routines and choreography.

In the field of dance, assessing aerobic capacity using objective measures such as heart rate monitors and ergometers is crucial for tailoring training programs at an individual level [4]. Determining an individual’s cardiovascular and respiratory capabilities is important for effectively managing the training load and focusing on achieving positive aerobic outcomes during the preparatory period. However, evaluating ‘maximal’ aerobic levels in dance presents challenges due to its non-competitive nature and the predetermined intensity by a choreographer [6]. Traditional objective methods for assessing aerobic performance, such as running- or cycling-based ergometers, are not well-suited for dance as they are costly, difficult to administer, and do not replicate the intricate movements involved in dance. Furthermore, dancers may experience biomechanical constraints during running or walking, stemming from limited dorsiflexion movements in the ankle and the highly developed turn out of the feet, leading to potential stress on the knee and hip joints [7]. When running, dancers often experience discomfort and pain in the shin area and uneven force distribution between tendons, ligaments, and muscles (especially in the calf area), leading to a higher risk of injuries or strain-related musculoskeletal problems [8]. To address these limitations, previous studies have endeavored to develop on-field aerobic tests specifically tailored for dance. A systematic review by Tiemens et al. [4] identified several common cardiorespiratory fitness test protocols in dance, including the Aerobic Power Index (API) [9], the Ballet-Specific Aerobic Fitness Test (B-DAFT) [10], the Dance Aerobic Fitness Test (DAFT) [11], the High-Intensity Dance Performance Fitness Test (HIDT) [12], and the Seifert Assessment of Functional Capacity for Dancers (SAFD) [13].

Although an effort has been made to adequately predict aerobic outcomes derived from objective methods from dance-specific testing protocols [9,10,11,12,13], most previous studies have correlated the VO_2_max and maximal heart rate (HRmax) from a single device [10,11]. Also, the agreement between correlation coefficients for the VO_2_max and associated factors ranged from −0.12 [13] to 0.91 [11], limiting the ‘true’ validity in dancesport athletes. Finally, the available tests have been primarily developed for ballet or contemporary dancers [10,11,12,13] at the individual level, while the validity properties in ballroom dance couples engaging in standard dance styles have yet to be investigated. Ballet and contemporary dancers have different training routines and movement patterns compared to the standard dance practitioners [14]. Also, ballet dancers have been shown to have an even lower accumulated fat mass %, which often goes below 10%, while ballroom style dancers tend to maintain their fat mass % between 10 and 20%, respectively [15]. Standard male and female ballroom dancers tend to have higher average VO_2_max values [15], compared to ballet or contemporary dancers [10,11]. This would imply that higher levels of the VO_2_max and peak heart rate (HRpeak) are generally needed to maintain performance at a high level and to cope with training and competition demands [15]. From a sport-specific point of view, ballroom dancesport athletes who come from modern dance typically develop aerobic capacity and maximal performance at different rates, while athletes from other endurance activities tend to increase their aerobic capacities linearly throughout their career [15]. On the other hand, by comparing the maximal aerobic capacity of dancers to other endurance sport athletes, the average VO_2_max is 48.0 mL * kg^−1^ * min^−1^, while it ranges between 55.0 and 77.0 mL * kg^−1^ * min^−1^ in swimmers, middle- and long-distance runners, rowers, and triathlon athletes [1]. Interestingly, the VO_2_max in dancers is similar to sedentary behaviors (≈44.0 mL * kg^−1^ * min^−1^), highlighting that the endurance in dancesport athletes is only developed throughout dance routines, while the development-oriented endurance training has yet to be investigated [1]. Thus, aerobic capacities and field-based tests in these athletes cannot be generalized to other dance styles, due to different physiological profiles and training/performance regimes during the preparatory and competition periods [1,4]. By examining the regular training and performance by using a specific test to assess aerobic capacity, coaches and choreographers would be able to monitor and track both cardiovascular and respiratory demands of different standard dance styles and could adjust the training volume to tackle physiological enhancements.

Because of the shortcomings from previously developed and validated dance protocols, the primary purpose of this study was to develop and validate a newly established Ballroom Aerobic Test (BAT) protocol specifically designed for dance couples performing in various standard dance styles. A secondary purpose was to establish the typical error of measurement and Bland–Altman agreement between the protocols. Based on the available evidence, we hypothesized that the BAT protocol would yield large to very large criterion validity properties against treadmill-based methods (KF1 and Bruce protocols) for measuring aerobic capacity.

## 2. Materials and Methods

### 2.1. Experimental Approach

In this study, a within subject test–retest sub-study was utilized to examine the validity properties of the BAT in standard dancesport athletes. This study was part of a project entitled ‘Construction and validation of a test protocol to assess aerobic capacity in dancesport athletes from standard dance styles.’ The project was divided into four segments to examine (i) reliability and usefulness; (ii) validity; (iii) sensitivity; and (iv) pragmatic validity of the BAT. For the purpose of this study, two standard progressive treadmill tests (the KF1 and Bruce protocols) were assessed to determine aerobic capacity, followed by the BAT protocol one week later. The testing procedure was initiated during the preparatory period of dancesport athletes. Measurements were conducted by the same experienced researcher to avoid possible measurement errors. During testing, the participants wore their standard dance equipment (light T-shirt, tights, dance shoes). The air temperature conditions of indoor facility and laboratory were controlled to be between 22 °C and 24 °C with a humidity of ≈55%.

### 2.2. Study Participants

Through a public call between April 2025 and June 2025, a convenience sample of twenty-four standard dancesport athletes (12 dance couples; age: 20.4 ± 3.9 years; height: 172.1 ± 8.7 cm; weight: 60.1 ± 9.4 kg) with 8.2 ± 3.4 years of training and competing experience participated in the study. All participants were members of certified dance clubs who competed at national and internation levels. The inclusion criteria to enter the study were as follows: (i) being free from any kind of injury, acute or chronic illness, and disease, confirmed by a certified dance association doctor; (ii) age range between 16 and 35 years; and (iii) complete both measurements for validity properties of the BAT protocol. The exclusion criteria included having dance experience < 5 years, suffering from musculoskeletal or any other injury that precluded them from entering and completing the testing procedures, being without a training partner, which could not mimic conditions from competition, and not being able to complete the field- and laboratory-based protocols. The a priori power analysis calculated by the G*Power software ver. 3.1.9.7 [16] showed that by setting the input parameters of a two-tailed α < 0.05, a minimum required correlation between the two methods at *r* > 0.69, and statistical power of 1 − β = 0.95, the required total sample size was n = 16. Because of a possible drop-out rate, we enlarged the sample size by 50% to *n* = 24. Before the study began, all participants had signed a written informed consent form to participate in the study and to use data solely for scientific purposes. All procedures in this study were anonymous and in accordance with the Declaration of Helsinki [17]. The Ethical Committee of the Faculty of Kinesiology approved this study on 29 April 2020 (ethical code number: 77/2020).

### 2.3. The BAT Protocol

To assess the level of aerobic capacity of dancesport athletes, we constructed a field-based BAT protocol. The protocol was standardized by 5 standard dance styles with the following order: (i) English waltz; (ii) slow fox; (iii) tango; (iv) Viennese waltz; and (v) quick step. Every dance style had multiple levels of dancing that were progressively linked, where the speed of dancing (tempo) was defined by individual beats per minute (bpm). The English waltz consisted of 6 levels, danced in 3/4 time in the range of or 75–110 bpm. The slow fox included 5 levels, danced in 4/4 time in the range of 117–145 bpm. The tango had 5 levels, danced in 4/4 time in the range of 152–180 bpm. The Viennese waltz was carried out through 3 levels, danced in 3/4 time in the range of 187–201 bpm. Finally, the quick step consisted of 6 levels, danced in 4/4 time in the range of 208–243 bpm. At each level, dance couples had to perform standard dance figures for 30 s at a defined speed without the melody (Table 1), while the time was cumulatively summed from the beginning of the BAT protocol. The speed expressed in beats progressively increased throughout the protocol by 7 bpm. The initial speed of the dance protocol was set at 75 bpm. Participants danced to a pre-defined dance tempo and rhythm by following the metronome. The elements for each dance style were basic and easy to perform, with the freedom of dance couples to create their own choreography. The duration of the BAT protocol was cumulatively added up and each pair danced until exhaustion.

### 2.4. The MetaMax^®^ 3B Outcomes

To examine the reliability properties of the BAT protocol, we used the MetaMax^®^ 3B (CORTEX Biophysik GmbH, Leipzig, Germany), a reliable and valid portable breath-by-breath ventilatory and metabolic measurement system specifically designed for field- and laboratory-based testing [18]. The device was composed of two parts designed to be worn on the chest. By using an electrochemical cell and an infrared analyzer, the MetaMax^®^ 3B was able to calculate O_2_ and CO_2_ concentrations based on standard metabolic equations [19]. Based on manufacturer’s recommendations and previous studies [17], the system was turned on for at least 20 min and calibrated prior to the testing. The calibration process included adjusting the gas analyzers by using reference gas values of O_2_ (14.97%), CO_2_ (4.96%), and N_2_ (±0.02%) and volume with a standardized 3 L syringe (5530 series, Hans Rudolph, Inc., Kansas City, MO, USA). The MetaMax^®^ 3B software generated data regarding absolute (L * min^−1^) and relative VO_2_max (mL * kg^−1^ * min^−1^) and associated variables including respiratory exchange ratio of CO_2_ produced to O_2_ consumed during metabolism (RER), expiratory ventilation as the total volume of air inhaled or exhaled per min (VE; L * min^−1^), tidal volume as the volume inhaled or exhaled during a normal breath (VT; L), ventilatory equivalent for O_2_ (VE/VO_2_) and CO_2_ (VE/VCO_2_) as determinants of breathing efficiency, and dead space to tidal volume ratio (VD/VT), which indicated the air that failed to participate in O_2_ and CO_2_ exchange process during breathing.

The testing procedure indicated that both male and female dancers (one dance couple) were simultaneously measured with the MetaMax^®^ 3B in order to simulate conditions from competitions in real-life settings. Although the order of trial was fixed, where dance couples first had to be tested for the BAT protocol and 48h after with the KF1 and 72 h after the KF1 protocol with the Bruce protocol in laboratory conditions, the possibility of a learning effect was vastly diminished. Each dance couple was tested individually to other dance couples for both field-and laboratory-based protocols. Also, they were told to keep the dance protocols and procedures to themselves and not to unveil them to the next dance couple. The BAT protocol was performed in an environment specific to dance, regarding the floor and dance attire that needed to be worn, while the KF1 and Bruce protocols were examined in laboratory conditions.

### 2.5. The KF1 and Bruce Testing Protocols

VO_2_max and associated ventilatory and metabolic parameters were assessed by a breath-by-breath pulmonary gas exchange system (Quark b^2^, COSMED, Roma, Italy) during an incremental treadmill test (KF1 and Bruce). KF1 is a standard test for the assessment of aerobic and anaerobic energy capacity [20]. The starting speed of the KF1 was 3 km * h^−1^ at a slope of 1% (continuous) for a duration of 2 min, after which the speed increased by 0.5 km * h^−1^ at the end of the third min and after that every 30 s until volitional exhaustion. During recovery after each test protocol, the subjects walked at 5 km * h^−1^ for 3 min.

In the Bruce protocol, the participants started exercising at a treadmill speed of 2.7 km * h^−1^ and an incline of 10% gradient for 3 min. Workloads (speed and inclination) were subsequently increased each 3 min period in a simultaneous way until volitional exhaustion was reached [21]. Both protocols generated equal cardiorespiratory data as the MetaMax^®^ 3B, which were filtered and averaged on a 5 s basis.

### 2.6. Data Analysis

Shapiro–Wilk’s (S-W) test was used to assess data normality. Data were checked for the critical *W* values, Q-Q plot inspection and the homogeneity of the variance was confirmed using the Leven test. If a calculated *W* value was larger than tabulated *W* value, this denoted that we did not reject the null hypothesis. All variables included in further analysis came from a hypothetical normal distribution, so data were presented as means and standard deviations (SDs) with 95% confidence interval limits (95% CI). To examine whether there was a couple dependence present during the BAT protocol, we utilized a linear-mixed effects model (LMM) with random intercept for couples and showed that there was no dependence effect within each couple for VO_2_max, as our primary outcome of the study (*F* = 1.533, *p* = 0.244). Differences between each protocol were examined with repeated measures analysis of variance (RM ANOVA). The magnitude of the change between the two measurements (the BAT protocol vs. KF1 and Bruce protocols) was examined with a standardized mean difference and the following effect sizes (ESs) proposed by Hopkins et al. [22]: trivial (ES < 0.2), small (0.2 < ES < 0.5), moderate (0.5 < ES < 0.8), large (0.8 < ES < 1.6), and very large (ES > 1.6). Degree of coherence between ventilatory and metabolic parameters of the BAT protocol vs. KF1 and Bruce protocols was assessed using Pearson’s product–moment correlation (*r*). Correlation values denoted association between variables and tests as small (*r* = 0.1–0.3), moderate (*r* = 0.3–0.5), large (*r* = 0.5–0.7), very large (*r* = 0.7–0.9), and almost perfect (*r* = 0.9–1.0) [23]. Since VO_2_max is considered as one of the most important determinants of performance in dancesport athletes [1,4,5], a Bland–Altman plot was created to present a graphical visualization of the agreement between the BAT and KF1/Bruce protocols for relative VO_2_max. In brief, the difference between the BAT and KF1 (BAT and Bruce) protocol was calculated on the *y*-axis, while the average of the two measurements was presented on the *x*-axis. To be able to determine the mean difference ideally close to (0) and the upper and lower limits of agreement (±1.96 SD), we created three horizontal lines. Based on data representation, we were able to (i) determine visual agreement between the two methods; (ii) identify possible bias where the mean difference was significantly different from 0; (iii) assess the presence of magnitude of random error; and (iv) establish the limits of agreement to confirm whether the differences between the two methods were clinically meaningful [24]. Finally, we calculated Lin’s concordance coefficient of correlation (*ρc*), an analysis which tests both precision and accuracy of bivariate pairs of observations relative to a ‘gold standard’ or another set of data. According to Altman [25], the cut-off points for ‘poor’ vs. ‘excellent’ agreement were <0.20 and >0.80. In addition, a Passing–Bablok regression analysis was considered as a non-parametric technique for comparing the BAT with the KF1 and Bruce protocols to observe a potential error in both measurements [26]. All statistical analyses were performed in SPSS v27.0 software (IBM, Armonk, NY, USA), with an alpha level set a priori at *p* < 0.05 to denote statistical significance.

## 3. Results

### 3.1. Primary Outcomes

According to the objective termination criteria of an RER ≥ 1.10, of the 24 participants, the proportion of the participants who reached this cut-off was 20.8% for the BAT protocol and 45.8% and 37.5% for the KF1 and Bruce protocols. Basic descriptive statistics of the study participants are presented in Table 2. No significant differences between the KF1 and BAT nor between the Bruce and BAT protocols were observed for the absolute VO_2_max (*F*_1,3_ = 0.206; *p* = 0.816; *η*^2^*_p_* = 0.02) and relative VO_2_max (*F*_1,3_ = 1.502; *p* = 0.245; *η*^2^*_p_* = 0.12), RER (*F*_1,3_ = 2.272; *p* = 0.094; *η*^2^*_p_* = 0.24), VE (*F*_1,3_ = 1.956; *p* = 0.165; *η*^2^*_p_* = 0.15), VT (*F*_1,3_ = 2.833; *p* = 0.065; *η*^2^*_p_* = 0.31), VE/VO_2_ (*F*_1,3_ = 2.514; *p* = 0.104; *η*^2^*_p_* = 0.19) and VE/VCO_2_ (*F*_1,3_ = 2.737; *p* = 0.087; *η*^2^*_p_* = 0.20), and VD/VT (*F*_1,3_ = 2.878; *p* = 0.078; *η*^2^*_p_* = 0.21). ESs showed trivial to small changes. Large to very large correlations were calculated between the KF1 and BAT protocols for absolute VO_2_max (*r* = 0.88; 95% CI = 0.75–0.95; *p* < 0.001) and relative VO_2_max (*r* = 0.88; 95% CI = 0.76–0.95; *p* < 0.001), RER (*r* = 0.78; 95% CI = 0.59–0.90; *p* < 0.001), VE (*r* = 0.86; 95% CI = 0.62–0.96; *p* < 0.001), VT (*r* = 0.75; 95% CI = 0.57–0.87; *p* < 0.001), VE/VO_2_ (*r* = 0.81; 95% CI = 0.55–0.91; *p* < 0.001) and VE/VCO_2_ (*r* = 0.78; 95% CI = 0.65–0.94; *p* < 0.001), and VD/VT (*r* = 0.70; 95% CI = 0.40–0.87; *p* < 0.001). Large to very large correlations were obtained between the Bruce and BAT protocols for absolute VO_2_max (*r* = 0.87; 95% CI = 0.72–0.95; *p* < 0.001) and relative VO_2_max (*r* = 0.85; 95% CI = 0.71–0.93; *p* < 0.001), RER (*r* = 0.76; 95% CI = 0.50–0.91; *p* < 0.001), VE (*r* = 0.79; 95% CI = 0.50–0.94; *p* < 0.001), VT (*r* = 0.83; 95% CI = 0.71–0.91; *p* < 0.001), VE/VO_2_ (*r* = 0.80; 95% CI = 0.64–0.92; *p* < 0.001) and VE/VCO_2_ (*r* = 0.82; 95% CI = 0.75–0.94; *p* < 0.001), and VD/VT (*r* = 0.74; 95% CI = 0.44–0.89; *p* < 0.001).

Lin’s analysis indicated an excellent concordance between the KF1 and BAT protocols for the absolute VO_2_max (*ρc* = 0.93; 95% CI = 0.84–0.97) and relative VO_2_max (*ρc* = 0.92; 95% CI = 0.82–0.97), RER (*ρc* = 0.86; 95% CI = 0.68–0.94), VE (*ρc* = 0.92; 95% CI = 0.82–0.97), VT (*ρc* = 0.85; 95% CI = 0.66–0.94), VE/VO_2_ (*ρc* = 0.88; 95% CI = 0.71–0.95) and VE/VCO_2_ (*ρc* = 0.88; 95% CI = 0.71–0.95), and VD/VT (*ρc* = 0.81; 95% CI = 0.57–0.92). Similarly, the data showed an excellent concordance between the Bruce and BAT protocols for the absolute VO_2_max (*ρc* = 0.93; 95% CI = 0.83–0.97) and relative VO_2_max (*ρc* = 0.90; 95% CI = 0.78–0.96), RER (*ρc* = 0.83; 95% CI = 0.62–0.93), VE (*ρc* = 0.88; 95% CI = 0.73–0.95), VT (*ρc* = 0.87; 95% CI = 0.70–0.94), VE/VO_2_ (*ρc* = 0.89; 95% CI = 0.73–0.95) and VE/VCO_2_ (*ρc* = 0.88; 95% CI = 0.73–0.95), and VD/VT (*ρc* = 0.84; 95% CI = 0.63–0.93).

### 3.2. Secondary Outcomes

**Figure 1:**
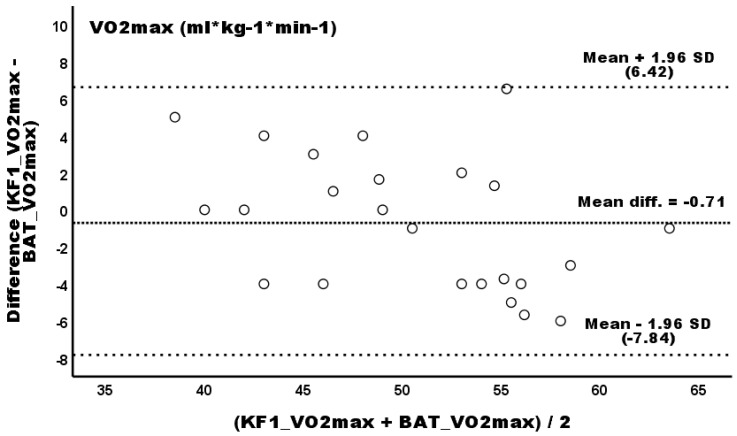
Bland–Altman plot of differences between the BAT and KF1 protocols (*y*-axis) and the average of the two measurements (*x*-axis) for relative VO_2_max.

Figure 1 shows the Bland–Altman scatterplot of the VO_2_max agreement between the BAT and KF1 protocols. The mean difference between the two methods yielded a value of −0.71, indicating no measurement bias (*p* > 0.05). Although a negative linear trend was observed, a linear regression analysis showed no potential proportional bias (*β* = −0.24; 95% CI −0.46–0.02; *p* = 0.066), with a standard error for the estimate (SEE) of 3.36 mL * kg^−1^ * min^−1^. The linear regression equation to predict the relative VO_2_max from the KF1 protocol was as follows: VO_2_max_KF1_ (mL * kg^−1^ * min^−1^) = −4.305 + (1.100 * VO_2_max_BAT_) (*R^2^* = 0.77; *p* < 0.001). The slope coefficient between the BAT and KF1 protocols was *b* = 1.625 with a 95% CI (0.90, 2.25), while the intercept coefficient was *a* = −18.43 with a 95% confidence interval (−64.65, 42.13). According to Passing and Bablok [26], if one was included in the CI for the slope and zero was included for the intercept, the BAT procedure yielded similar measurements to the KF1 procedure.

**Figure 2:**
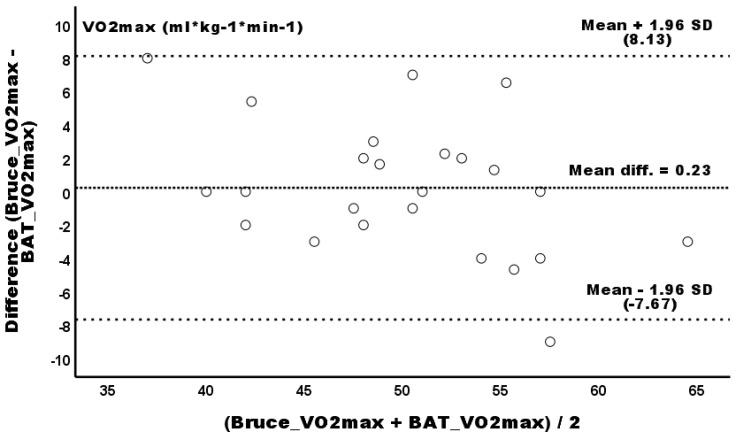
Bland–Altman plot of differences between the BAT and Bruce protocols (*y*-axis) and the average of the two measurements (*x*-axis) for relative VO_2_max.

The Bland–Altman scatterplot of the VO_2_max agreement between the BAT and Bruce protocols is presented in Figure 2. The mean difference between the two methods yielded a value of 0.23, indicating no measurement bias (*p* > 0.05). Similarly to the BAT-KF1, a negative linear trend between the BAT and Bruce protocols was observed; however, no potential proportional bias was shown (*β* = −0.26; 95% CI −0.51–0.15; *p* = 0.087). The generated SEE of the VO_2_max was 3.75 mL * kg^−1^ * min^−1^. Similarly to the KF1 protocol, the linear regression equation to predict the relative VO_2_max from the Bruce protocol was as follows: VO_2_max_BRUCE_ (mL * kg^−1^ * min^−1^) = −3.996 + (1.075 * VO_2_max_BAT_) (*R^2^* = 0.72; *p* < 0.001). The slope coefficient between the BAT and KF1 protocols was *b* = 1.10 with a 95% CI (0.80, 1.30), while the intercept coefficient was *a* = −1.05 with a 95% confidence interval (−10.17, 9.90), indicating that the BAT protocol yielded similar measurements to the Bruce protocol.

## 4. Discussion

The main purpose of this study was to develop and examine the criterion validity and Bland–Altman agreement of the BAT protocol against the KF1 and Bruce laboratory protocols in dancesport athletes performing in standard dance styles. Findings suggest that the ventilatory and metabolic parameters derived from the MetaMax^®^ 3B portable gas analyzer during the BAT protocol correlate as large to very large with the KF1 and Bruce aerobic outcomes. The level of agreement calculated from the Bland–Altman plots for the relative VO_2_max shows no systematic nor proportional biases between the two methods with the SEE of 3.36 mL * kg^−1^ * min^−1^ (KF1) and 3.75 mL * kg^−1^ * min^−1^ (Bruce).

To the best of the authors’ knowledge, this is the first study examining the development and validity of a progressive test to assess aerobic capacity in standard dancesport athletes. Our results should be interpreted in light of previous studies. To date, several progressive field-based tests to assess the level of aerobic capacity in dance have been proposed and validated against more objective methods. For example, a study by Wallmann et al. [9] showed that the API submaximal exercise fitness test correlated well with the VO_2_max (*r* = 0.89 and 0.90), with a technical error of measurement (TEM) of a power output = 0.07 W * kg^−1^ and VO_2_max = 0.71 mL * kg^−1^min^−1^. Another dance protocol designed for ballet dancers indicated that the mean squared root (s_w_^2^) for the test was 5.01, with a SEE of 6.20 mL * kg^−1^min^−1^ [10]. One of the most common fitness protocols to examine aerobic capacity is the DAFT, a five-stage 4 min progressive test [10]. In a study by Wyon et al. [11], data generated from the portable telemetric gas analyzer (Cosmed K4 b2, Italy) indicated that the HRmax and VO_2_max correlated very strongly for the test (*r* = 0.91; SEE = 5.60 b * min^−1^). Similar observations were noted for the HIDT dance protocol, where five participants (n = 5) undertook both the treadmill and dance tests to estimate the VO_2_max [12]. During the treadmill protocol, the mean VO_2_max was 46.40 ± 3.60 mL * kg^−1^min^−1^, and during the HIDT, the VO_2_max value was 51.00 ± 6.60 mL * kg^−1^min^−1^. A pairwise comparison between the treadmill and dance protocols showed no significant differences in VO_2_max (*p* = 0.100) [12]. The same study also showed that the mean % of the HRmax was significantly lower in the HIDT compared to the treadmill test (97.50 vs. 101.00%, *p* = 0.020), while the validity of blood lactate values were high (HIDT = 6.10 ± 1.90 mmol*L^−1^ vs. treadmill = 6.30 ± 1.70 mmol * L^−1^, *p* = 0.860) [12]. The most recent SAFD test to evaluate the functional capacity of collegiate dancers revealed large to very large correlations with the peak treadmill test for the relative VO_2_max (*r* = 0.78), HRmax (*r* = 0.85), blood lactate (*r* = 0.72), and rate of perceived exertion (RPE; *r* = 0.84), while trivial to moderate correlations were found for time (*r* = 0.60) and the RER (*r* = −0.12) [13].

Although the existing progressive dance protocols to assess aerobic capacity exhibited satisfactory validity properties against objective measures [9,10,11,12,13], our newly developed BAT protocol yielded high agreements with the treadmill test following two protocols (KF1 and Bruce). The incremental difference and the novelty between our and other dance protocols lies in the fact that it is specifically designed for dance couples who engage in standard dance styles. This is very important, because both individuals need to cope with each other during the rehearsal/training or competition. This would suggest that their aerobic capacity needs to be at equal level in order to keep up with the partner. Despite the fact that the VO_2_max tends to be low in dancesport athletes, compared to other athletes [4,5,6], the intention of the BAT protocol is to enhance the aerobic system and to physiologically prepare for stress during training and/or performance. Dance movements in standard dance styles are often characterized by sway components and the interplay between rises and falls, which are considered key elements for preserving or increasing functional performance in competitive dancesport athletes [27]. Moreover, the complexity of figures and faster pace during training and competition in standard dance styles is not comparable to ballet or contemporary dance styles, where the movements are often performed in static conditions, emphasizing esthetic rather than functional components of dance. Therefore, the BAT protocol can serve as an avenue for screening and tracking aerobic capacity within the narrow niche of standard style dance athletes.

The practical application of the BAT protocol is reflected in the development of a completely new model for measuring aerobic capacity and an algorithm for estimating the VO_2_max in standard dances under the conditions of the sport-specific activity. This enables a realistic assessment of physical and energy loads specific to the activity, in comparison with the existing tests for assessing aerobic capacity. Since standard dance styles have been often categorized as high-intensity physical activities that need aerobic and anaerobic energy systems to perform [28], the newly established dance protocol would be able to increase aerobic capacity and to optimize dance training. Indeed, correct and timely diagnostic procedures for measuring and/or assessing energy capacities in dance are needed to achieve primary goals and positive trends towards sports success [1,4].

This study is not without limitations. First, a cross-sectional design prevents us from confirming a causal relationship between field- and laboratory-based testing protocols for assessing aerobic capacity. Second, we did not include the HRmax nor blood lactate parameters as indicators of aerobic and anaerobic capacities, limiting our findings to data generated from the MetaMax^®^ 3B and treadmill devices. Previous studies have shown a strong relationship between HRmax and VO_2_max [10,11] and adequate sensitivity in HRmax and blood lactate changes following the dance protocol [13]. Third, a relatively small sample size might have underpowered the validity properties of the BAT protocol. Although we performed a sample size calculation (for more information, please see the ‘Study Participants’ section), we cannot exclude the lack of statistical power. Fourth, we failed to examine the rate of perceived exertion (RPE) and collect blood samples for lactate concentrations following the BAT, KF1, and Bruce protocols. These physiological measures have been constantly used as potential markers for determining fatigue and should be considered in further research in dancesport athletes. Fifth, the BAT protocol was measured with the MetaMax^®^ 3B, while the treadmill tests used COSMED Quark b^2^. The difference in devices could introduce systematic errors independent of the protocol. Finally, the BAT protocol was not cross-validated in other athletes from different dance styles (ballet, contemporary, or Latin American), limiting its applicability. Nevertheless, the authors suggest that the BAT protocol is a valid training and performance tool for measuring aerobic capacity and associated factors in dancesport athletes from standard dance styles.

## 5. Conclusions

In summary, this study shows that the BAT protocol has a strong to excellent criterion validity against the KF1 and Bruce protocols. Also, the Bland–Altman analysis indicated that the SEE for the KF1 and Bruce protocols against the BAT protocol was 3.36 mL * kg^−1^ * min^−1^ and 3.75 mL * kg^−1^ * min^−1^. The advantage of the BAT protocol is that it simulates classical movements from five dance styles and mimics competition conditions. The current findings have provided evidence of its validity properties and potential guidelines for monitoring and tracking ventilatory and metabolic parameter changes under different training conditions. The authors suggest that this test can be implemented within the dance training program in preparatory and competitive periods, as it gives important and valid information about the agreement between field and laboratory aerobic performance variables among standard dancesport athletes.

## Figures and Tables

**Table 1:** The BAT dance protocol for every dance style.

Dance Styles	Stage 1	Stage 2	Stage 3	Stage 4	Stage 5	Stage 6
*English waltz*						
Beats	75	82	89	96	103	110
Time	30 s	60 s	1.5 min	2 min	2.5 min	3 min
*Slow fox*						
Beats	117	124	131	138	145	
Time	3.5 min	4.0 min	4.5 min	5.0 min	5.5 min	
*Tango*						
Beats	152	159	166	173	180	
Time	6.0 min	6.5 min	7.0 min	7.5 min	8.0 min	
*Viennese waltz*						
Beats	187	194	201			
Time	8.5 min	9.0 min	9.5 min			
*Quick step*						
Beats	208	215	222	229	236	243
Time	10.0 min	10.5 min	11.0 min	11.5 min	12.0 min	12.5 min

Time = cumulative time from BAT start.

**Table 2:** Basic descriptive statistics, mean difference (diff.), and ES between the BAT protocol against the KF1 and Bruce protocols.

Study Variables	KF1	Bruce	BAT	Absolute Diff. (95% CI)		Relative Diff. (%)		ES	
	Mean ± SD	Mean ± SD	Mean ± SD	KF1–BAT	Bruce–BAT	KF1–BAT	Bruce–BAT	KF1–BAT	Bruce–BAT
**Absolute VO_2_** (L * min^−1^)	3.05 ± 0.74	3.05 ± 0.75	3.00 ± 0.65	−0.05 (−0.19–0.10)	−0.05 (−0.20–0.11)	1.64%	1.64%	0.07 (trivial)	0.07 (trivial)
**Relative VO_2_** (mL * kg^−1^ * min^−1^)	50.92 ± 7.47	49.98 ± 7.55	50.21 ± 5.96	−0.71 (−2.24–0.83)	0.23 (−1.47–1.93)	1.39%	0.46%	0.11 (trivial)	0.03 (trivial)
**RER**	1.09 ± 0.05	1.08 ± 0.04	1.07 ± 0.06	−0.02 (−0.03–0.02)	−0.01 (−0.02–0.02)	1.83%	0.93%	0.36 (small)	0.20 (small)
**VE** (L * min^−1^)	110.21 ± 28.43	110.43 ± 28.75	104.20 ± 26.37	−6.01 (−12.22–0.20)	−6.23 (−13.83–1.37)	5.45%	5.64%	0.22 (small)	0.23 (small)
**VT** **(L)**	1.93 ± 0.42	1.98 ± 0.54	1.77 ± 0.36	−0.16 (−0.28–−0.04)	−0.22 (−0.54–−0.28)	8.29%	11.11%	0.41 (small)	0.46 (small)
**VE/VO_2_**	33.65 ± 3.82	33.85 ± 3.46	32.93 ± 2.93	−0.71 (−1.67–0.24)	−0.91 (−1.78–0.04)	2.11%	2.69%	0.21 (small)	0.29 (small)
**VE/VCO_2_**	30.90 ± 3.83	30.16 ± 2.67	31.04 ± 3.51	0.14 (−0.89–1.17)	0.88 (−0.03–1.73)	0.45%	2.92%	0.04 (trivial)	0.28 (small)
**VD/VT**	0.15 ± 0.03	0.14 ± 0.03	0.15 ± 0.02	0.003 (−0.005–0.01)	0.009 (−0.001–0.02)	2.00%	6.43%	0.01 (trivial)	0.39 (small)

## Data Availability

The data that support the findings of this study are available upon reasonable request from the corresponding author. The data are not publicly available due to privacy or ethical restrictions.
